# Isolation of Chicken Intestinal Glial Cells and Their Transcriptomic Response to LPS

**DOI:** 10.3390/biology15030225

**Published:** 2026-01-25

**Authors:** Jie Chen, Wenxiang Zhang, Xingxing Tian, Feng Zhang, Chunsheng Xu

**Affiliations:** College of Animal Science and Technology, Shihezi University, Shihezi 832003, China; 18381680727@163.com (J.C.); z302729814@163.com (W.Z.); 15186769141@163.com (X.T.); 17509072722@163.com (F.Z.)

**Keywords:** chicken, EGC, LPS, transcriptome

## Abstract

Current research on glial cells has primarily focused on central nervous system glial cells (CNS glia), with relatively fewer studies on EGCs. Given the critical role of EGCs in maintaining intestinal homeostasis and neural function, this study aimed to investigate their immunomodulatory effects under inflammatory conditions. Primary EGCs were isolated and an inflammatory model was established by treatment with lipopolysaccharide (LPS). Following LPS induction, cellular samples were collected for transcriptomic analysis to identify differentially expressed genes. The analysis revealed that 88 genes were significantly altered, with 60 upregulated and 28 downregulated. Through Gene Ontology (GO) classification, Kyoto Encyclopedia of Genes and Genomes (KEGG) pathway mapping, and protein–protein interaction (PPI) network analysis, several key regulatory genes were identified: chemokine-related genes (*IL8L2*, *IL8L1*, *CCL4*, *CCL5*, and *CX3CL1*); negative feedback regulation-related genes (*TNFAIP3* and *ZC3H12A*); homeostasis-maintaining genes (*C1QB* and *LY86*); and arachidonic acid metabolism-related genes (*PTGS2* and *GGT2*). Under LPS stimulation without impairing EGC viability, EGCs may recruit immune cells by regulating the aforementioned genes. Additionally, arachidonic acid and its metabolites likely play important regulatory roles in EGC-mediated immunomodulation. These findings provide new theoretical insights and potential targets for further elucidating the pathogenesis of intestinal inflammation and developing targeted therapies.

## Simple Summary

Studying enteric glial cells (EGCs) in chickens extends beyond elucidating the functions of a single cell type. Instead, it serves as a central gateway to understanding how the avian enteric nervous system coordinates digestive, immune, and neural functions. From maintaining intestinal barrier integrity and regulating immune responses to promoting tissue repair, EGCs play a pivotal role in gut homeostasis. This research not only deepens the mechanistic understanding of these processes but also provides a scientific basis for optimizing livestock and poultry production.

## 1. Introduction

The intestine is the largest immune organ, constantly interacting with food antigens, microorganisms, and other stimuli [1]. Enteric glial cells (EGCs), a major component of the enteric nervous system, share morphological and functional similarities with astrocytes in the central nervous system and express unique markers such as glial fibrillary acidic protein (GFAP) and S100 calcium-binding protein B (S100B) [2,3]. Despite sharing a suite of core functions as part of the glial cell family, EGCs and central nervous system Glial Cells (CNS glia) reside in distinct organ systems, resulting in notable differences in their subtypes, functional roles, and local microenvironments [4]. Studies have demonstrated that EGCs are critical for regulating and maintaining intestinal barrier function; a reduction in their number or functional impairment can lead to intestinal barrier dysfunction, thereby triggering inflammatory diseases [5,6]. This regulatory role is partially mediated by various secreted factors, including glial cell line-derived neurotrophic factor (GDNF), substance P, neurotrophins, and transforming growth factor-β1 (TGF-β1), all of which participate in the modulation of mucosal barrier function to varying degrees [7]. EGCs engage in intimate crosstalk with intestinal immune cells and modulate the maintenance of intestinal immune homeostasis via cytokine-mediated signaling pathways [8]. Upon intestinal injury or under specific in vitro culture conditions, EGCs can reactivate the transcriptional programs of early enteric nervous system progenitors, thereby undergoing differentiation into mature neurons [9]. Notably, EGCs express toll-like receptor 4 (TLR4), a pattern recognition receptor for LPS [10]. In the intestine, LPS serves as both a key component of the microbiota and a critical molecule in host immune and metabolic regulation [11], with its behavior and effects influenced by intestinal barrier function, microbial composition, and host immune status [12]. LPS exerts dual roles in the intestine: on one hand, appropriate levels of LPS contribute to maintaining intestinal homeostasis and immune regulation [13]; on the other hand, excessive LPS—resulting from intestinal barrier disruption or dysbiosis—may induce inflammatory responses, leading to conditions such as inflammatory bowel disease and metabolic syndrome [14]. Given that LPS can function as both a microbial signal and a potential inflammatory trigger, and considering the emerging evidence for the role of EGCs in gut barrier and immune regulation, investigating how LPS may directly influence EGC function is of significant interest. However, the specific effects and underlying mechanisms of LPS on EGCs, particularly in non-mammalian species such as chickens, remain largely unexplored. To explore this, we established an inflammatory model using an optimized concentration of LPS and performed mRNA sequencing analysis.

## 2. Materials and Methods

### 2.1. Cell Isolation and Purification

Fertilized embryonated eggs from SPF (Specific Pathogen Free) White Leghorn chickens were purchased from Shandong Haotai Laboratory Animal Breeding Co., Ltd. (Haotai, Jinan, China) and incubated in our laboratory until day 18 of development. At this stage, intestinal tissues were aseptically collected from the embryos. The intestinal tissues were rinsed with PBS, minced into fragments, and digested with Collagenase II (Solarbio, Beijing, China) at 37 °C for 1 h. The digested mixture was filtered through a 70 μm cell strainer (Biosharp, Hefei, China) and centrifuged at 850× *g* for 5 min. After discarding the supernatant, the cell pellet was resuspended and subjected to a second digestion step using 0.25% trypsin (Solarbio) and 20 U/mL Deoxyribonuclease I (Solarbio) at 37 °C for 7 min [15]. The enzymatic reaction was terminated by adding DMEM/F12 complete medium (Gibco, Waltham, MA, USA) supplemented with 2.5% chicken serum (Solarbio), 7.5% fetal bovine serum (Tianhang, Hangzhou, China), and 1% triple antibiotics (Biosharp). The cell suspension was then gently pipetted to obtain to dissociate cell clusters, re-filtered through a 70 μm cell strainer, and centrifuged again to collect the pellet.

The cell pellet was resuspended in complete medium and seeded into T75 flasks pre-coated with 1 mg/mL Poly-L-Lysine (Solarbio). To enrich for EGCs, the flasks were incubated in a 5% CO_2_ incubator at 38.5 °C for 15 min to allow for selective adherence of fibroblasts. The supernatant, enriched with non-adherent EGCs, was transferred to new pre-coated T75 flasks for continued culture. This differential plating process was repeated five times. After 24 h of culture in T75 flasks without Poly-L-Lysine coating, first remove the original medium and gently wash with PBS to eliminate suspended non-target cells (e.g., neurons, T cells, and B cells). Subsequently, add 1.5 U/mL Dispase II (Solarbio) for 5 min to further remove any residual fibroblasts [16]. Finally, the cells were cultured for 48 h in DMEM/F12 medium supplemented with 1% triple antibiotics, 1% G-5 Supplement (Procell, Wuhan, China) [17], 0.25% chicken serum, and 0.75% fetal bovine serum prior to experimental use.

### 2.2. Immunofluorescence Staining for EGCs

Enteric glial cells and fibroblasts were identified using immunofluorescence staining for glial fibrillary acidic protein (GFAP) [7] and collagen type I alpha 1 chain (COL1A1) [18].

Cells were seeded onto coverslips and cultured for 24 h. The coverslips were rinsed three times with 0.01 mmol/L phosphate-buffered saline (PBS) for 5 min each, then fixed with PBS containing 4% paraformaldehyde (Biosharp) at room temperature for 15 min, followed by three additional 5 min rinses with PBS. After permeabilization with 0.2% Triton X-100 (Saitong, Beijing, China), the cells were blocked with goat serum (Solarbio, Beijing, China) for 1 h, and then incubated overnight at 4 °C with rabbit anti-GFAP antibody (1:500 dilution; Affinity Biosciences, Cincinnati, OH, USA) and mouse anti-COL1A1 antibody (1:500 dilution; Proteintech, Wuhan, China). Following three 5 min PBS rinses, the coverslips were incubated with FITC-conjugated goat anti-rabbit IgG secondary antibody (1:50 dilution; Zhongshan Jinqiao, Beijing, China) and TRITC-conjugated goat anti-mouse IgG secondary antibody (1:50 dilution; Zhongshan Jinqiao, Beijing, China) at room temperature for 1 h in the dark. After another three 5 min PBS rinses, ready-to-use anti-fluorescence quenching DAPI (Solarbio, Beijing, China) was added dropwise under dark conditions before mounting. Images were captured using a fluorescence microscope (Soptop, Ningbo, China). For cell counting, three random fields of view per coverslip were analyzed using ImageJ software (version 1.54g; http://imagej.org, accessed on 24 November 2025).

### 2.3. CCK8 and Quantitative Real-Time PCR Validation (qRT-PCR) of the Inflammation Model

To establish an inflammatory model of EGCs, we selected 10.0 μg/mL LPS as the reference concentration based on previous studies [19]. EGCs were stimulated with LPS at gradient concentrations (0 [control], 5, 10, 20, 40, 80, and 160 μg/mL) for 24 h. Cell viability was assessed using the CCK-8 Cell Proliferation and Cytotoxicity Assay Kit (Solarbio, Beijing, China) following the manufacturer’s instructions. After a 3 h incubation, absorbance was measured at 450 nm.

Based on the cell viability results, a concentration of 10 μg/mL LPS was selected for subsequent model validation. EGCs were then stimulated with this concentration, and the mRNA expression levels of inflammatory cytokines tumor necrosis factor-α (*TNF-α*) and interleukin-6 (*IL-6*) were detected by qRT-PCR. Significant upregulation of *TNF-α* and *IL-6* is considered a key indicator of successful establishment of the EGC inflammatory model. The detailed experimental procedures were as follows: Total RNA was isolated from cell samples using the TransZol Up Plus RNA Kit (TransGen Biotech, Beijing, China). For cDNA synthesis, the HiScript IV RT SuperMix for qPCR reverse transcription kit (Vazyme, Nanjing, China) was used. Primers (YOCON, Beijing, China) were designed using NCBI (https://www.ncbi.nlm.nih.gov/, accessed on 24 November 2025). qPCR was performed with the ChamQ Universal SYBR qPCR Master Mix (Vazyme, Nanjing, China) under the following conditions: initial denaturation at 94 °C for 30 s, followed by 40 cycles of denaturation at 94 °C for 5 s and annealing/extension at 60 °C for 30 s. All amplification reactions were run in triplicate for each gene. Relative quantification of gene-specific expression was calculated using the 2^−ΔΔCt^ method, with glyceraldehyde-3-phosphate dehydrogenase (GAPDH) as the internal reference gene.

### 2.4. Extract Total RNA for Transcriptomic Analysis

Total RNA was isolated from cell samples using the TransZol Up Plus RNA Kit (TransGen Biotech, Beijing, China) according to the manufacturer’s instructions. RNA integrity was assessed using the RNA Nano 6000 Assay Kit on a Bioanalyzer 2100 system (Agilent Technologies, Santa Clara, CA, USA). RNA concentration and purity were measured using a NanoDrop spectrophotometer (Thermo Fisher Scientific, Wilmington, DE, USA), with acceptable purity defined by A260/A280 ratios between 1.8 and 2.0 and A260/A230 ratios greater than 2.0.

### 2.5. Library Construction and Data Processing

cDNA library construction and RNA sequencing (RNA-seq) were performed by Bioprofile Co., Ltd. (Shanghai, China); six sample libraries were sequenced on the NovaSeq platform. Sequencing data were filtered using Fastp (version 1.0.1) to remove reads with adapters at the 3^′^ end and those with an average quality score below Q20, and clean reads were aligned to the chicken reference genome (GCF_000002315.6_GRCg6a) using HISAT2 (version 2.0.5).

### 2.6. Differential Gene Expression and Functional Enrichment Analysis

Differential gene expression between the LPS-treated and control groups was analyzed using DESeq2 (version 1.20.0). Differentially expressed genes (DEGs) with |log2FoldChange| > 1 and *p* < 0.05 were selected for exploratory analyses. GO terms and KEGG pathways with *p* < 0.05 were defined as significantly enriched.

### 2.7. Protein–Protein Interaction Analysis

Protein–protein interaction (PPI) networks of DEGs identified by RNA sequencing were constructed using the STRING database (version 10.5; https://string-db.org/, accessed on 24 November 2025) with a confidence threshold set to >0.4 [20]. This threshold facilitates capturing a broader range of potential interactions, making it suitable for subsequent exploratory analyses. The PPI networks were visualized and subjected to topological analysis using Cytoscape software (version 3.9.1). Node degrees were calculated to identify hub genes.

### 2.8. qRT-PCR Validation

To verify the reproducibility and reliability of RNA-seq data, seven genes were randomly selected for qRT-PCR analysis. The experimental method was performed as described above. Primer sequences are listed in Table 1.

### 2.9. Statistical Analysis

All results were based on at least three independent biological replicates. Intergroup statistical analyses were performed using unpaired *t*-tests or one-way analysis of variance (ANOVA) with GraphPad Prism 10.1.2 (GraphPad Software, San Diego, CA, USA). Error bars represent the mean ± standard deviation (SD), and a ** *p* value < 0.05 ** was considered statistically significant. Significance was denoted as follows: ns, *p* > 0.05; *, *p* ≤ 0.05; **, *p* ≤ 0.01; ***, *p* ≤ 0.001.

## 3. Results

### 3.1. Isolation and Purification of EGCs

**Figure 1:**
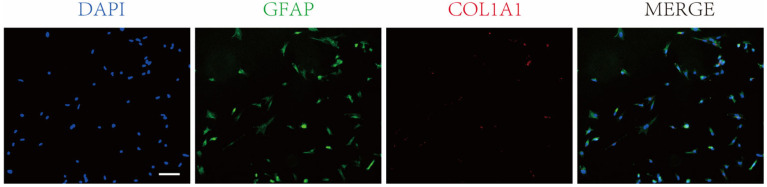
Immunofluorescence staining for GFAP and COL1A1 confirmed that the cultured cells were EGCs. Scale bar (applicable to all images) = 100 μm.

We successfully isolated primary EGCs from the intestines of chicken embryos, with a purity exceeding 90% (Figure 1). Recording-type cell culture images are shown in Supplementary Figure S1. The statistical data can be found in Table S1.

To preliminarily characterize the molecular profile of our cultures, we performed RNA-seq analysis (Figure S2). The transcriptomic data revealed high expression of *GJA1* and the stromal gene *COL1A1*. While *GJA1* is broadly expressed across tissues, its protein product is particularly enriched in EGCs within the gut, providing an initial clue for cell-type assignment [21]. Interestingly, the concurrent upregulation of *COL1A1* suggested a potentially activated glial state, as activated glia are known to elevate stroma-associated genes [22]. However, this transcriptional signature appeared inconsistent with the negligible COL1A1 protein accumulation observed in subsequent immunofluorescence assays—a discrepancy that may result from rapid collagen secretion or post-transcriptional regulation. In parallel, transcript levels of lineage-specific markers for other cell types, including canonical EGCs markers (*S100B*, *GFAP*), neuronal (*RBFOX3*), epithelial (*VIL1*), Paneth cell (*LYZ*), and notably the fibroblast-specific marker S100 Calcium Binding Protein A4 (*S100A4*), remained uniformly low. The minimal expression of *S100A4* provided strong evidence against significant fibroblast contamination. Together, these data present a complex signature: high *GJA1* aligns with intestinal *EGCs*, while elevated *COL1A1* contrasts with low off-lineage markers and diverges from protein-level detection. This underscores that transcript abundance does not always correspond to functional protein localization, particularly under in vitro conditions. Therefore, definitive cell identification required protein-level validation. Indeed, immunofluorescence analysis confirmed robust *GFAP* expression, providing key evidence for the presence of enteric glial cells in our cultures.

### 3.2. Establishment of Inflammatory Models

**Figure 2:**
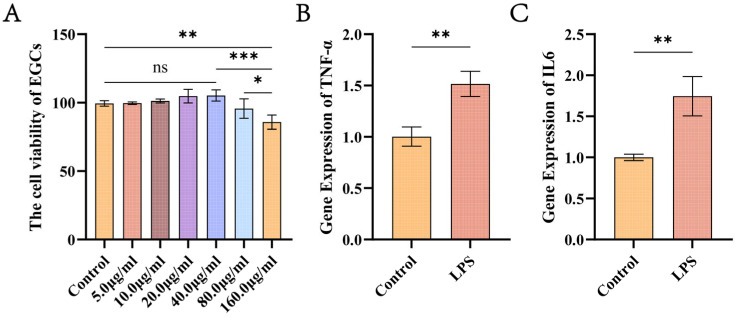
Stimulation of EGCs with 5–160 μg/mL LPS (**A**). Relative expression levels of inflammatory factors *TNF-α* (**B**) and *IL-6* (**C**) in EGCs after 24 h stimulation with 10 μg/mL LPS. Significance was denoted as follows: ns, *p* > 0.05; *, *p* ≤ 0.05; **, *p* ≤ 0.01; ***, *p* ≤ 0.001.

The results of the Cell Counting Kit-8 (CCK-8) assay showed that the cell viability in the 10.0 μg/mL LPS-treated group was approximately 100%, with no significant difference compared to the control group (Figure 2). This indicated that LPS at this concentration had no obvious effect on cell viability. qRT-PCR results further demonstrated that stimulation with LPS at the same concentration for 24 h successfully upregulated the expression of inflammatory factors *TNF-α* and *IL-6* in EGCs, thereby successfully establishing an inflammatory model.

### 3.3. Summary of Raw RNA-Seq Read Data

To investigate the global transcriptional response of chicken EGCs to LPS-induced inflammation, RNA-seq-based comparative analysis was performed on the transcriptomes of cells from the saline control group and LPS-treated group. RNA-seq results for the six cell samples are summarized in Table 2. The number of raw reads ranged from 40.94 million to 48.90 million. After filtering out low-quality reads, contaminants, and other artifacts from the raw data, the total number of clean reads ranged from 40.30 million to 48.13 million. Q20 scores exceeded 98%, indicating high sequencing quality, with GC content of clean reads ranging from 45.75% to 46.09%. The overall mapping rate was between 94.84% and 95.25%.

### 3.4. Differentially Expressed Genes Analysis

**Figure 3:**
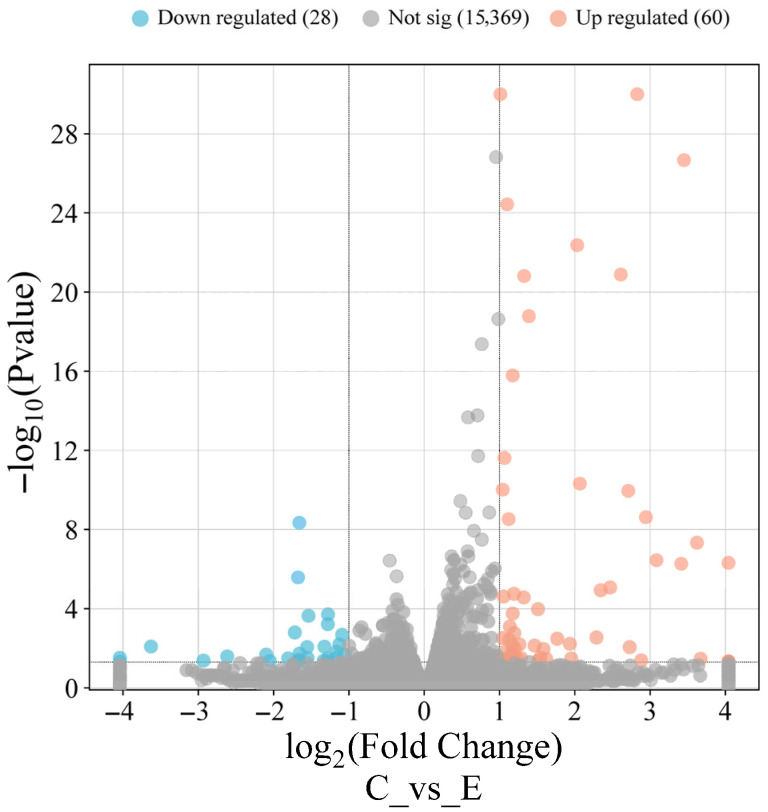
Volcano plot of differentially expressed genes (DEGs) in EGCs under inflammatory stimulation. Genes meeting the criteria of *p* < 0.05 and |log2 FC| ≥ 1 are defined as significantly DEGs. Red dots represent upregulated genes, blue dots represent downregulated genes, and gray dots represent non-significant genes. The *x*-axis and *y*-axis of the volcano plot show log2 fold change and −log10 *p* value, respectively, between the control group (C, *n* = 3) and LPS-treated group (E, *n* = 3).

A volcano plot was used to visualize the global distribution of DEGs (Figure 3). In total, 88 DEGs were identified in EGCs under inflammatory conditions, including 60 upregulated and 28 downregulated genes. Table 3 lists the top 20 DEGs with padj < 0.05 and |log_2_FC| ≥ 1.

### 3.5. GO and KEGG Pathway Analysis of DEGs

**Figure 4:**
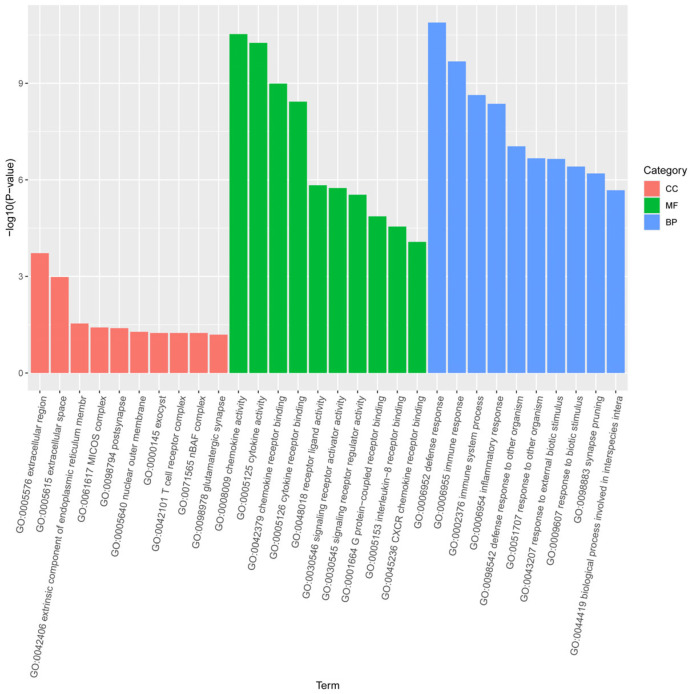
GO enrichment analysis. The bar chart illustrates the top 10 significantly enriched GO terms from the Biological Process (BP), Molecular Function (MF), and Cellular Component (CC) categories for the 88 DEGs identified in LPS-treated EGCs. Enrichment significance is measured by −log10(*p*-value).

The functions and pathways of the 88 DEGs were evaluated using GO and Kyoto Encyclopedia of KEGG pathway analyses. GO analysis categorized DEGs into biological processes (BP), molecular functions (MF), and cellular components (CC), revealing enrichment in 493 GO terms (Figure 4). Within the BP category, 424 GO terms were significantly enriched, with the top 5 being defense response, immune response, immune system process, inflammatory response, and defense response to other organism. In the MF category, 64 GO terms were significantly enriched, with the top 5 including chemokine activity, cytokine activity, chemokine receptor binding, cytokine receptor binding, and receptor ligand activity. For the CC category, 5 GO terms were significantly enriched, with the top 5 being extracellular region, extracellular space, extrinsic component of endoplasmic reticulum membrane, MICOS complex, and postsynapse. The 10 most significant GO terms are illustrated in (Figure 4).

KEGG pathway analysis identified 8 significantly enriched pathways: Cytokine-cytokine receptor interaction, Toll-like receptor signaling pathway, Cytosolic DNA-sensing pathway, NOD-like receptor signaling pathway, Influenza A, Arachidonic acid metabolism, Necroptosis, and RIG-I-like receptor signaling pathway (Table 4). The up-regulated and down-regulated gene sets are included in Supplementary Figures S3 and S4. Detailed information on GO and KEGG can be found in Supplementary Materials Tables S2 and S3.

### 3.6. PPI Analysis

**Figure 5:**
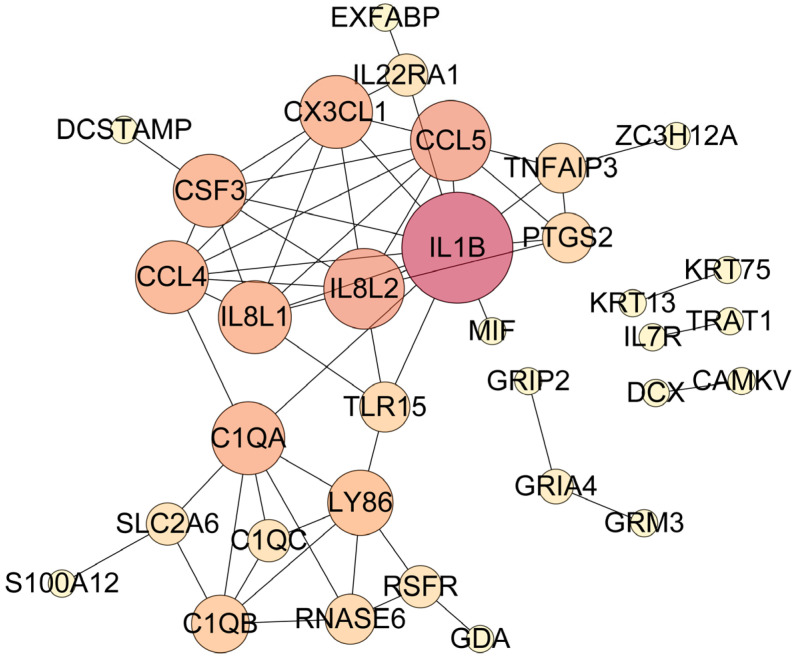
PPI network of differentially expressed genes in EGCs upon LPS stimulation. The size and color of each circle correspond to the degree value of each gene.

In this study, we used PPI analysis to explore the potential interactions between proteins encoded by DEGs. A total of five networks were identified, including one large network (Figure 5). The largest network contained multiple chemokines, and its genes were annotated in the KEGG pathway (*CCL4*, *CCL5*, *IL1B*, *IL8L1*, *CSF3T*, *CX3CL1*, *IL7R*, *IL8L2*, *TNFAIP3*, *PTGS2*) (Table 4). One of the other four networks included *GRIA4*, which was among the top 20 upregulated and downregulated differentially expressed genes (DEGs) (Table 3).

### 3.7. Verify RNA Sequencing Results via qRT-PCR

**Figure 6:**
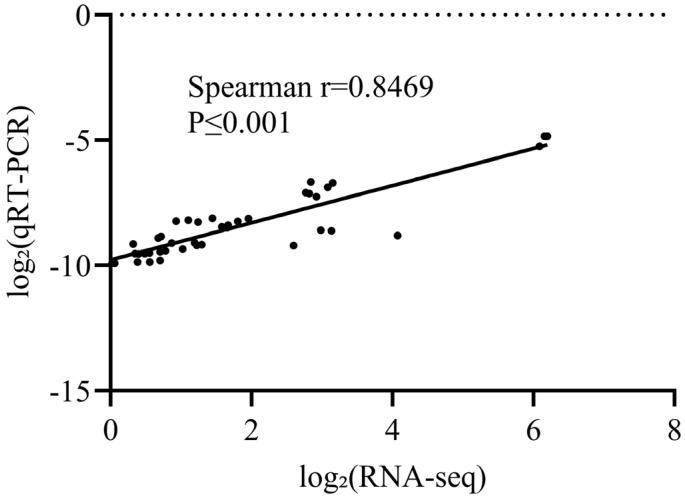
Validation of RNA-seq results by RT-qPCR.

Seven genes with high degree values were selected from the PPI network for qRT-PCR validation (Figure 6), including 5 upregulated transcripts (*IL8L2*, *CSF3*, *IL1B*, *GRIA4*, and *CX3CL1*) and 2 downregulated transcripts (*C1QA*, *LY86*). All genes showed consistent expression trends between qRT-PCR and RNA-seq (Spearman’s r = 0.8469, *p* ≤ 0.001). These results support the reliability and accuracy of the RNA-seq data in this study.

## 4. Discussion

### 4.1. Chemokine Storm and Immune Cell Recruitment

In this study, we observed significant upregulation of multiple chemokine family members, including *IL8L2*, *IL8L1*, *CCL4*, *CCL5*, and *CX3CL1*, strongly indicating the initiation of a chemokine storm and subsequent robust recruitment of inflammatory cells. Astrocytes and microglia can produce IL8L2 (IL-8) in response to inflammatory stimuli such as LPS [23]; its primary function is to recruit and activate neutrophils. Upregulated *IL8L2* triggers downstream signaling pathways such as MAPK and PKB by activating CXCR1/2 receptors, amplifying the inflammatory cascade [24]. The concurrent upregulation of IL8L1, which shares similar functions, further enhances these chemotactic signals [25]. Additionally, the marked upregulation of *CCL4* and *CCL5* suggests that activated glial cells are actively recruiting peripheral immune cells to infiltrate the CNS parenchyma; notably, these factors also play critical roles in regulating neuronal autophagy and neurodegenerative processes [26].

The upregulation of *CX3CL1* carries unique dual significance in this context. Its membrane-bound form, predominantly expressed on neurons and astrocytes, maintains microglia in a quiescent surveillance state through binding to the specific receptor CX3CR1 on microglia [27]. However, under inflammatory conditions, astrocytes upregulate *CX3CL1*, which is then cleaved by proteases such as ADAM10 to release its soluble form, s*CX3CL1* [28]. Soluble *CX3CL1* potently chemoattracts CX3CR1-expressing microglia and monocytes. Thus, the upregulation of *CX3CL1* marks a critical functional shift from a homeostatic “calming” signal to an inflammatory “alert” and “recruitment” signal. Furthermore, *PTGS2* (*COX-2*), a key effector enzyme in inflammatory responses, is upregulated to catalyze the conversion of arachidonic acid into potent inflammatory mediators (prostaglandins), further driving the inflammatory process [29].

### 4.2. Amplification of Inflammatory Responses and Negative Feedback Regulation

In the mechanism of inflammatory amplification, the upregulation of *S100A12* is particularly critical. When cells are damaged or stressed, extracellularly released S100A12 acts as an endogenous ligand to activate TLR4 and RAGE receptors, thereby triggering the NF-κB pathway and inducing the production of more proinflammatory cytokines (including IL-8), forming a self-amplifying positive feedback loop [30]. Its upregulation in glial cells indicates that these cells not only respond to initial stimuli but also actively generate endogenous signals to sustain and intensify the inflammatory state.

Concurrently, we observed activation of key negative feedback regulatory mechanisms. Upregulation of *TNFAIP3* and *ZC3H12A* represents the critical negative feedback regulators negative feedback nodes in inflammatory signaling pathways. TNFAIP3 is a zinc finger protein with both deubiquitinase and E3 ubiquitin ligase activities, whose core function is to inhibit NF-κB signaling through multiple mechanisms [31,32]. Notably, TNFAIP3 expression itself is induced by NF-κB [33], and its functional deficiency leads to abnormal proliferation of microglia [34]. Similarly, ZC3H12A (Regnase-1) primarily exerts its ribonuclease activity to specifically degrade mRNA of proinflammatory cytokines such as *IL-6* and *IL-1β*, precisely terminating inflammatory signals at the post-transcriptional level [35]. Additionally, ZC3H12A has been reported to possess deubiquitinase activity, enabling it to inhibit signaling pathways such as JNK and NF-κB [36]. Like TNFAIP3, ZC3H12A expression is also induced by inflammatory signals (e.g., IL-1β) [37], forming another important negative feedback regulatory pathway. The coordinated upregulation of *TNFAIP3* and *ZC3H12A* in EGCs likely represents a core mechanism employed by EGCs to suppress excessive immune activation and maintain intestinal homeostasis.

### 4.3. Metabolic and Transport Function Remodeling

Immune activation requires substantial energy and biosynthetic precursors to support its functions. The upregulation of genes encoding related transporters reflects this metabolic reprogramming. SLC13A5 (a citrate transporter) mediates the uptake of extracellular citrate into cells, providing substrates for the tricarboxylic acid cycle, fatty acid synthesis, and production of inflammatory mediators such as prostaglandins. Upregulation of *SLC2A6* (a glucose transporter) ensures glucose uptake [38]. The coordinated upregulation of these two transporters collectively indicates that glial cells undergo energy and anabolic remodeling to meet the demands of their highly active inflammatory state.

Upregulation of *VNN1* leads to the production of cysteamine, which increases glutathione (GSH) levels and enhances cellular antioxidant capacity. Meanwhile, as the demand for coenzyme A (CoA) increases, cysteamine helps buffer reactive oxygen species (ROS) generated by elevated mitochondrial activity, thereby maintaining the homeostasis of energy metabolism. Additionally, the upregulation of *VNN1* indicates that cells are undergoing lipid metabolism remodeling to meet energy demands and support the synthesis of lipid inflammatory mediators (e.g., eicosanoids) [39].

### 4.4. Suppression of Cellular Homeostasis-Maintaining Functions

In contrast to the widespread upregulation of inflammation-related genes, the expression of several genes responsible for maintaining cellular homeostasis is repressed. C1QB (the B chain of complement C1q), typically produced by microglia [40], plays a key role in tagging redundant synapses to guide critical synaptic pruning by microglia [41]. The significant downregulation of *C1QB* strongly suggests that, under acute inflammatory conditions, microglia may suspend their elaborate homeostatic functions to redirect resources toward responding to danger signals.

Similarly, *LY86* encodes MD1, an auxiliary protein in TLR signaling pathways. Downregulation of *LY86* may reflect remodeling of TLR signaling to prevent excessive and sustained immune activation [42]. This could also indicate a shift in cellular responses from a broad-spectrum surveillance mode to a more focused reaction targeting specific threats.

### 4.5. Systemic Immune Responses Revealed by GO and KEGG Enrichment Analyses

Enrichment analysis of differentially expressed genes in chicken EGCs upon LPS stimulation showed significant enrichment in terms related to immune response (GO:0006954), cytokine activity (GO:0005125), neuronal synapse pruning (GO:0098883), and glutamatergic synapses (GO:0098978). These findings suggest that LPS-induced immune activation in EGCs is not limited to initiating inflammation but may directly mediate structural and functional remodeling of intestinal neural circuits. EGCs regulate inflammatory responses through secreted signaling molecules and potentially mediate synaptic remodeling, highlighting their critical role in intestinal immune-neural crosstalk. The results reveal that LPS-induced immune activation not only triggers inflammation but also likely reshapes neural function, emphasizing a dual role in maintaining intestinal homeostasis.

KEGG pathway analysis further uncovered a clear inflammatory cascade: danger signals are cooperatively recognized by pattern recognition receptors, which activate key transcription factors such as NF-κB/IRFs to drive the production of initial cytokines/interferons. Subsequently, the cytokine network is rapidly amplified, and local inflammatory responses are mediated through arachidonic acid metabolism. Cell death pathways such as necroptosis may clear damaged cells and release damage-associated molecular patterns (DAMPs), which in turn re-stimulate pattern recognition receptors, forming a positive feedback loop that sustains inflammation. Notably, arachidonic acid metabolites are key mediators of information exchange between the intestine and microbiota; their dysregulation may disrupt intestinal microbial balance, closely linking to disease pathogenesis [43,44,45].

### 4.6. Core Regulatory Hubs Identified by PPI Network Analysis

PPI network analysis highlighted IL1B as a central hub in the network. Notably, five highly clustered chemokines (*IL8L2*, *IL8L1*, *CCL4*, *CCL5*, *CX3CL1*) interact closely with *IL1B*. Previous studies have shown that *IL1B* can positively regulate chemokine expression and secretion via the NF-κB pathway [46]; conversely, certain chemokines (e.g., *CCL4* and *CCL5*) have been reported to inhibit ATP-induced *IL-1β* release [47]. These observations suggest that in EGCs, LPS may activate the NF-κB pathway to coordinate chemokine production through *IL1B* as a key hub, thereby activating immune responses. Concurrently, EGCs likely finely regulate IL-1β release via negative feedback regulators such as *TNFAIP3* and *ZC3H12A* [48,49], achieving dynamic balance between immune activation and intestinal homeostasis maintenance.

## 5. Conclusions

Our analysis demonstrates that chicken enteric glial cells (EGCs) undergo a functional activation upon LPS stimulation, shifting toward a proinflammatory state characterized by the orchestrated induction of a chemokine network (e.g., *IL8L2*, *CCL4*) and dynamic regulation of negative feedback hubs (e.g., *TNFAIP3*). This activated phenotype is further defined by the concurrent upregulation of immunomodulatory cytokines and altered expression of key genes critical for homeostasis maintenance (e.g., *C1QB*). Furthermore, our data reveal significant upregulation of the arachidonic acid metabolic pathway in EGCs under inflammatory conditions, suggesting a potential, novel role for EGCs in which arachidonic acid metabolites may mediate communication with the intestinal microbiota or immune cells to regulate intestinal microenvironmental homeostasis, a hypothesis that warrants further validation. One limitation of this study is that while the GFAP+ enteric glial cells isolated by this method constitute over 90% of the population, they do not fully reflect the diverse subtypes and proportions present in intact tissue; this simplified model cannot fully recapitulate the complex in vivo environment.

## Figures and Tables

**Table 1:** RT-qPCR primer sequences used in this study.

Gene	Accession No.	Primer Sequence (5^′^→3^′^)	Amplicon Size (bp)
*IL-6*	NM_204628.2	F:CGCCTTTCAGACCTACCTGG	181
		R:CTTCAGATTGGCGAGGAGGG	
*TNF-α*	NM_204267.2	F:CCCATCTGCACCACCTTCAT	115
		R:CGGAGGGTTCATTCCCTTCC	
*C1QA*	XM_046903298.1	F:ACAACAACAGCCGCAACATC	92
		R:CGGGATGGTGTTCACAGACA	
*CSF3*	NM_205279.2	F:GGAGGTGTGCTTCACTCAGAT	96
		R:ACCAACGTCGTGTGATTGGG	
*CX3CL1*	NM_001077232.2	F:GACTTCGACCTCAACCTCCG	94
		R:TGCACTGATTGTGTCCAGGG	
*IL8L2*	NM_205498.2	F:TGCTCTGTCGCAAGGTAGGA	183
		R:AAGCACACCTCTCTTCCATCC	
*GRIA4*	NM_001113186.2	F:AGCGTGCAAATAGGTGGTCT	184
		R:ACTGGGAGCAGAAGGCATTT	
*LY86*	NM_001004399.2	F:GAGGGACCAATCACACTGGG	84
		R:GGCGCGATCTTCGTTAGTCA	
*IL-1β*	NM_204524.2	F:GCCTGCAGAAGAAGCCTCG	210
		R:GGAAGGTGACGGGCTCAAAA	

**Table 2:** RNA sequencing readouts and mapping rates in intestinal glial cells of chickens.

Sample ^1^	Raw Reads	Clean Reads No.	Clean Reads Q20 ^2^ (%)	GC (%)	Total Mapping (%)
C1	41,807,344	41,147,602	98.42	46.03	95.25
C2	48,904,882	48,135,090	98.43	46.09	95.04
C3	40,949,830	40,308,530	98.43	45.89	94.99
E1	46,080,530	45,418,484	98.56	45.75	95.01
E2	46,756,418	46,109,596	98.62	45.91	95.00
E3	42,450,388	41,754,072	98.36	45.89	94.84

^1^ The control group (C1, C2, C3) and LPS-treated group (E1, E2, E3) each contained three biological replicates (*n* = 3). Each replicate originated from an independently conducted cell culture batch. ^2^ Q20 indicates the percentage of bases with a Phred value ≥ 20.

**Table 3:** Top 20 differentially expressed genes in the inflammation model.

Transcript	Gene	log2 Fold Change	Padj	Regulated
ENSGALG00000048781	*-*	4.041423199	0.000212522	up
ENSGALG00000005995	*SLC13A5*	3.62225265	2.55054 × 10^−5^	up
ENSGALG00000013993	*VNN1*	3.45021803	8.33094× 10^−24^	up
ENSGALG00000038995	*GRIA4*	3.413799508	0.000230763	up
ENSGALG00000043064	*EXFABP*	3.08453186	0.000166111	up
ENSGALG00000046160	*-*	2.943511802	1.59979× 10^−6^	up
ENSGALG00000026098	*IL8*	2.82922875	1.188 × 10^−118^	up
ENSGALG00000024272	*S100A12*	2.709680757	9.06647 × 10^−8^	up
ENSGALG00000001437	*NTM*	2.611209484	2.84729× 10^−18^	up
ENSGALG00000043603	*CCL5*	2.469783889	0.002588915	up
ENSGALG00000026768	*SLCO4C1*	2.344003557	0.003404077	up
ENSGALG00000011668	*IL8L1*	2.066816628	4.36738 × 10^−8^	up
ENSGALG00000040832	*CFD*	2.031691279	1.10911 × 10^−19^	up
ENSGALG00000030907	*CSF3*	1.511251987	0.021506119	up
ENSGALG00000034478	*CCL4*	1.390629303	2.84772 × 10^−16^	up
ENSGALG00000026663	*CX3CL1*	1.32715325	2.96985 × 10^−18^	up
ENSGALG00000004771	*C1QB*	−1.675751996	0.000910912	down
ENSGALG00000012801	*LY86*	−1.658468273	2.81508 × 10^−6^	down
ENSGALG00000027165	*RSFR*	−1.536722539	0.038655549	down
ENSGALG00000021395	*-*	−1.277397192	0.033578951	down

**Table 4:** EGCs genes and KEGG pathways potentially affected by LPS.

Pathway Term	Count	*p* Value	Gene Symbols ^1^
gga04060: Cytokine-cytokine receptor interaction	9	9.67 × 10^−8^	*SCYA4 ↑*, *CCL4 ↑*, *CCL5 ↑*, *IL1B ↑*, *IL8L1 ↑*, *CSF3 ↑*, *CX3CL1 ↑*, *IL7R ↑*, *IL8L2 ↑*
gga04620: Toll-like receptor signaling pathway	6	2.6549 × 10^−6^	*SCYA4 ↑*, *CCL4 ↑*, *CCL5 ↑*, *IL1B ↑*, *IL8L1 ↑*, *IL8L2 ↑*
gga04623: Cytosolic DNA-sensing pathway	4	3.1929 × 10^−5^	*SCYA4 ↑*, *CCL4 ↑*, *CCL5 ↑*, *IL1B ↑*
gga04621: NOD-like receptor signaling pathway	5	0.0004	*CCL5 ↑*, *IL1B ↑*, *IL8L1 ↑*, *IL8L2 ↑*, *TNFAIP3 ↑*
gga05164: Influenza A	4	0.0035	*IL8L1 ↑*, *CCL5 ↑*, *IL1B ↑*, *IL8 ↑*
gga00590: Arachidonic acid metabolism	2	0.0161	*GGT2 ↑*, *PTGS2 ↑*
gga04217: Necroptosis	3	0.0197	*TNFAIP3 ↑*, *IL1B ↑*, *H2A ↓*
gga04622: RIG-I-like receptor signaling pathway	2	0.0266	*IL8L1 ↑*, *IL8L2 ↑*

^1^ The upward and downward arrows, respectively, represent the genes that are upregulated and downregulated in intestinal glial cells under the action of LPS.

## Data Availability

The transcriptome sequencing data have been deposited in the National Center for Biotechnology Information (NCBI) under the BioProject accession number PRJNA1346664.

## Supplementary Materials

The following supporting information can be downloaded at: https://www.mdpi.com/article/10.3390/biology15030225/s1. Tables S1–S3. Figure S1. Recording-type cell culture images of EGCs cultured at different timepoints, eyepiece X10, objective X10; Figure S2. Expression profile of enteroglial cell-associated marker genes in cultured tissue; Figure S3. GO biological process enrichment analysis of DEGs. Upregulated gene enrichment results (A), Downregulated gene enrichment results (B), Enrichment results for all differentially expressed genes (C), Bar height represents enrichment factors; Figure S4. KEGG pathway enrichment analysis of differentially expressed genes. Enriched terms for up-regulated genes (A), Enriched terms foor down-regulated genes (B), Enriched terms for all differentially expressed genes (C).

## Abbreviations

The following abbreviations are used in this manuscript:

*C1QA*	Complement C1q subcomponent A
*C1QB*	Complement C1q subcomponent B
*C1QC*	Complement C1q subcomponent C
*CAMKV*	CaM kinase like vesicle associated
*CCL4*	C-C motif chemokine ligand 4
*CCL5*	C-C motif chemokine ligand 5
*CSF3*	Colony stimulating factor 3
*CX3CL1*	C-X-3-C motif chemokine ligand 1
*DCSTAMP*	Dendrocyte expressed seven transmembrane protein
*DCX*	Doublecortin
*EXFABP*	Extracellular fatty-acid-binding protein
*GDA*	Guanine deaminase
*GRIA4*	Glutamate ionotropic receptor AMPA type subunit 4
*GRIP2*	Glutamate receptor interacting protein 2
*GRM3*	Glutamate metabotropic receptor 3
*H2A*	Histone H2A
*IL1B*	Interleukin-1 beta
*IL22RA1*	Interleukin 22 receptor subunit alpha 1
*IL7R*	Interleukin-7 receptor
*IL8L1*	interleukin 8-like 1
*IL8L2*	interleukin 8-like 2
*KRT13*	Keratin 13
*KRT75*	Keratin 75
*LY86*	Lymphocyte antigen 86
*MIF*	Macrophage migration inhibitory factor
*NTM*	Protein CEPU-1
*PTGS2*	Prostaglandin-endoperoxide synthase 2
*RNASE6*	Ribonuclease A family member 6
*RSFR*	Leukocyte ribonuclease A-2
*S100A12*	S100 calcium-binding protein A12
*SCYA4*	C-C motif chemokine 4 homolog
*SLC13A5*	Solute carrier family 13 member 5
*SLC2A6*	Solute carrier family 2 member 6
*SLCO4C1*	Solute carrier organic anion transporter family member 4C1
*TNFAIP3*	Tumor necrosis factor alpha-induced protein 3
*TRAT1*	T-cell receptor associated transmembrane adaptor 1
*VNN1*	vanin 1
*ZC3H12A*	Zinc finger CCCH-type containing 12A
