# Supplementary material for: Isolation of Chicken Intestinal Glial Cells and Their Transcriptomic Response to LPS
Source: Biology (Basel). 2026 Jan 25;15(3):225. doi: 10.3390/biology15030225 (PMC12896624; doi:10.3390/biology15030225)
Supplement: Supplementary file 1 [file biology-15-00225-s001.zip › Supplementary Materials-Figures S1-S4.pdf]

Biology

Isolation of Chicken Intestinal Glial Cells and Their Transcriptomic Response to LPS

Jie Chen †, Wenxiang Zhang †, Xingxing Tian, Feng Zhang and Chunsheng Xu *

Supplementary Figure S1-S4

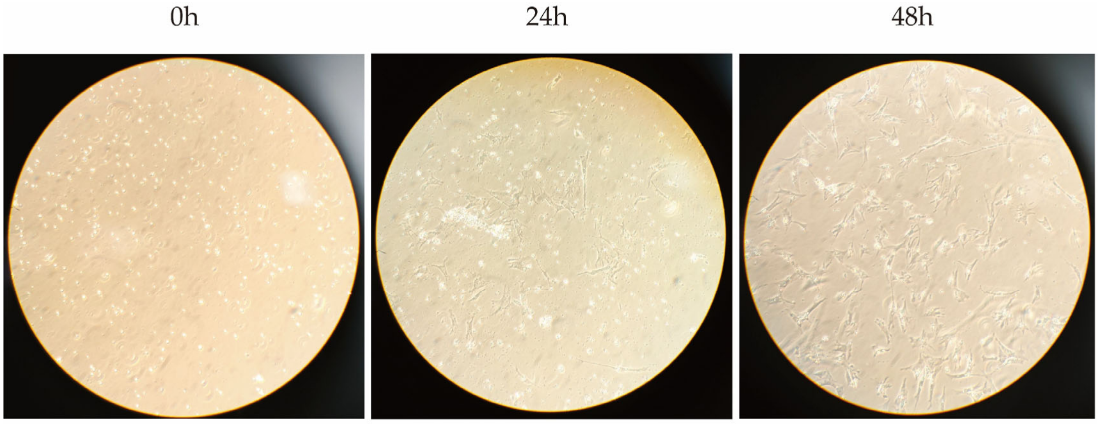

Figure S1. Recording-type cell culture images of EGCs cultured at different timepoints, eyepiece X10, objective X10.

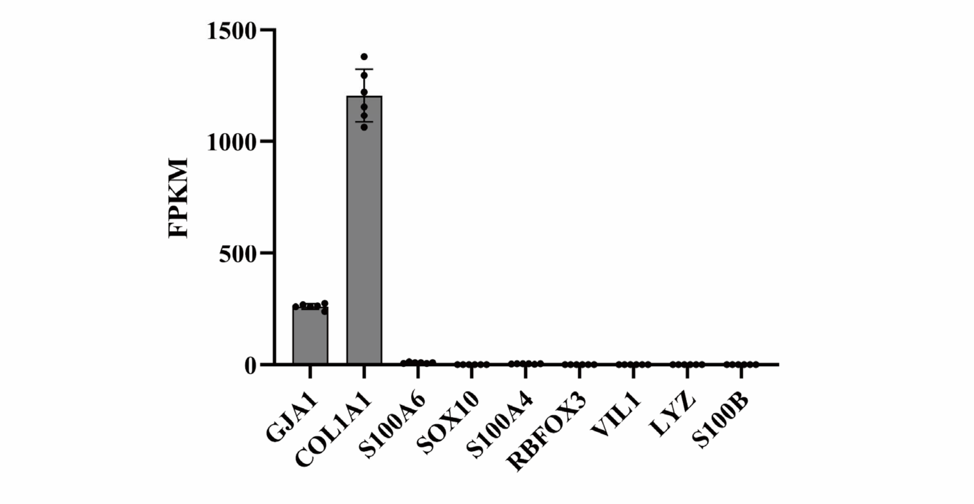

Figure S2. Expression profile of enteroglial cell-associated marker genes in cultured tissue.

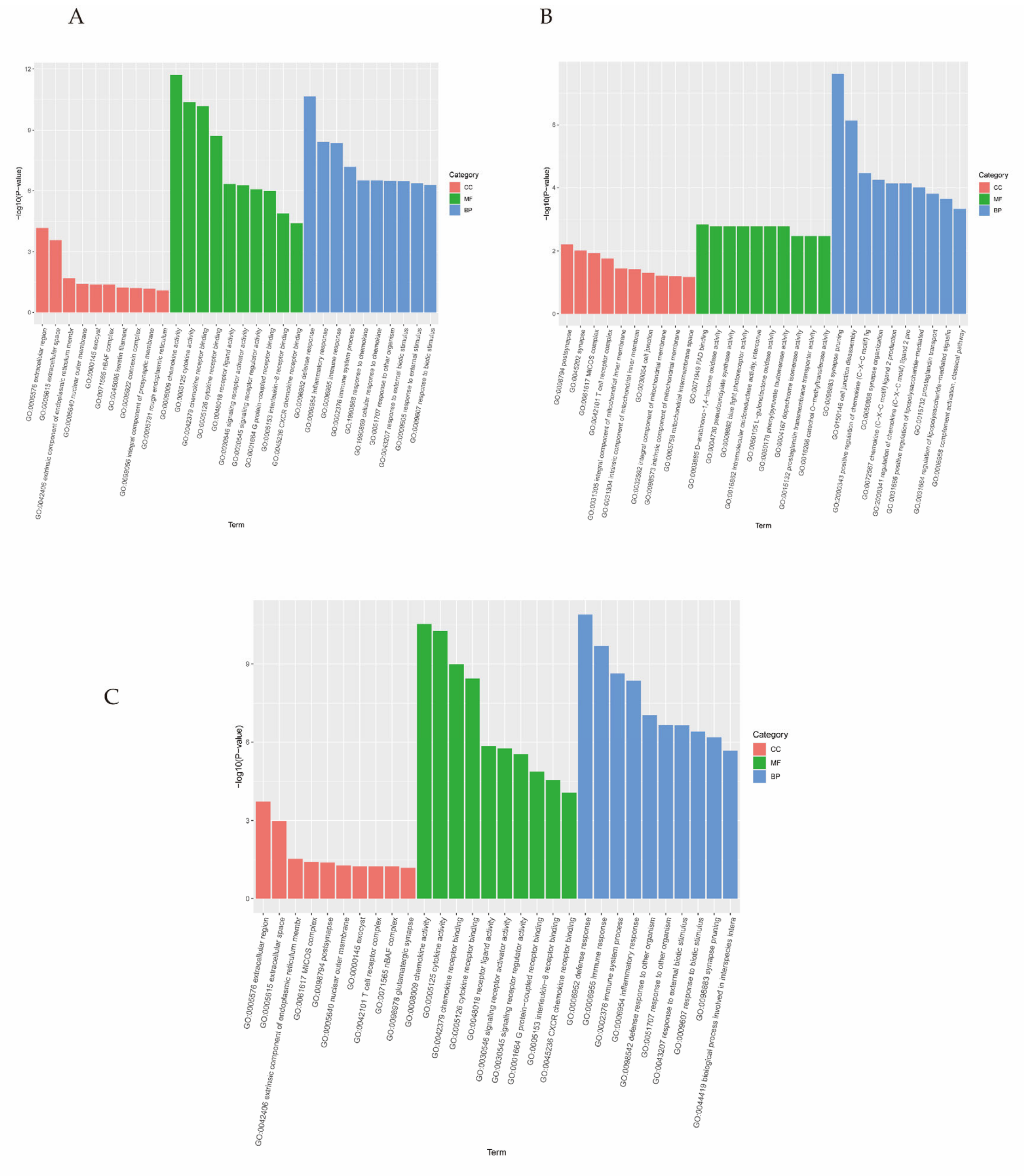

Figure S3. GO biological process enrichment analysis of DEGs. Upregulated gene enrichment results (A), Downregulated gene enrichment results (B), Enrichment results for all differentially expressed genes (C), Bar height represents enrichment factors.

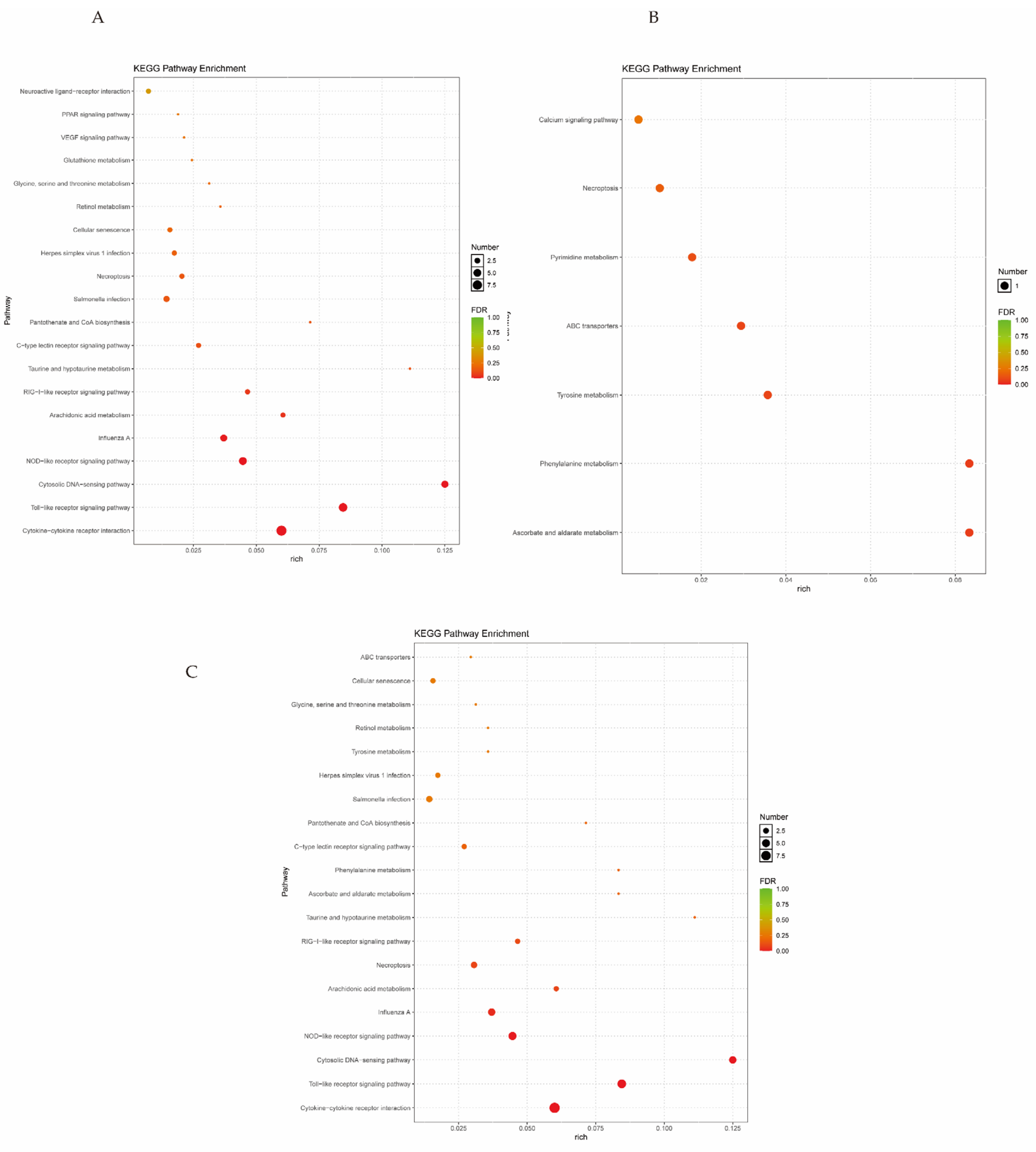

Figure S4. KEGG pathway enrichment analysis of differentially expressed genes. Enriched terms for up-regulated genes (A), Enriched terms foor down-regulated genes (B), Enriched terms for all differentially expressed genes (C).
